# Graded opsin co-expression along the butterfly retina fine tunes the spectral sensitivity of a colour-opponent cell across the visual field

**DOI:** 10.1007/s00359-025-01761-6

**Published:** 2025-09-26

**Authors:** Andrew Dang, Uroš Cerkvenik, Marko Ilić, Primož Pirih, Eva Debevc, Adriana D. Briscoe, Gregor Belušič

**Affiliations:** 1 Department of Ecology and Evolutionary Biology, University of California, Irvine, Irvine, CA USA; 2 Department of Biology, Biotechnical Faculty, University of Ljubljana, Večna Pot 111, 1000 Ljubljana, Slovenia; 3 Department for Chemistry and Physics of Materials, University of Salzburg, Jakob-Haringer-Str. 2a, 5020 Salzburg, Austria

**Keywords:** Opsin, Co-expression, Butterfly, Compound eye, Colour vision

## Abstract

Compound eyes deliver a vast stream of information to the tiny insect brains. To maximize the information content and minimize the redundancy of neural signals, insect eyes are built so to encode the relevant and filter out the unimportant elements of the visual environment. Terrestrial habitats have a predictable spatio-spectral structure, which can be matched by the distribution of photoreceptors with different spectral sensitivities across the retina. Here, we investigate the retinal organization of the nymphalid butterfly *Heliconius melpomene* using single-cell recordings, immunohistochemistry and eye shine imaging. The ventral retina is enriched with ommatidia, which contain red screening pigments that shape the spectral sensitivity of basal red receptors R9, while their long visual fibre photoreceptors R1&2, expressing a long-wavelength (L) opsin, are synaptically inhibited by R9 and directly participate in colour vision. These G + R– receptors frequently co-express the L opsin with the blue (B) or ultraviolet (U) opsin. U&L opsin-co-expressing R1&2 are scarce, while B&L co-expression is frequent in the ventral ommatidia and gradually diminishes towards the eye equator, where G + R– receptors express the L opsin only. In this region, G + R– receptors are further inhibited by blue-sensitive receptors. With electrophysiology matching immunohistochemistry, we reveal the fine tuning of spectral sensitivity of a single photoreceptor class across the dorso-ventral axis of the butterfly compound eye. Similar tuning is found in other nymphalid butterflies across the phylogeny, suggesting that this adaptation is ancestral and confers an advantage to those diurnal nymphalids, equipped with the cellular toolkit for colour vision in the red.

## Introduction

Insect compound eyes sample the visual space around the animal in virtually all directions, creating a neural image, which contains the information about the intensity, spectral composition and polarization of incident light (Land and Nilsson 2012). This stream of multidimensional information is delivered to the brain, which then drives various behaviours. The visual system imposes a large metabolic cost (Niven and Laughlin 2008), and this in turn drives evolutionary adaptations that minimize the processing costs by discarding or ignoring redundant information in the early stages of information processing, resulting in a process termed matched filtering (Wehner 1987; Warrant 2016). A notable example of a matched filter is the retinal mosaic, where photoreceptors with different spectral sensitivities are distributed across the retinal lattice semi-randomly, but with pronounced spectral sensitivity and spatial acuity gradients, and with localized specializations such as acute zones or the dorsal rim area (Land 1989; Wernet et al. 2015). The different architectures of the retinal mosaics are presumably suited to maximize the neural signal and minimize noise and information redundancy.

The visual world has a predictable spatial and spectral structure, known as the light field (Nilsson and Smolka 2021). For instance, the dorsal and ventral hemispheres of the light field of most terrestrial and aquatic habitats are information-rich in the short- and long-wavelength part of the spectrum, respectively (Zimmermann et al. 2018; Qiu et al. 2021). Consequently, the dorsal and ventral-viewing retinae are predominantly occupied with short- and long-wavelength photoreceptors. This arrangement has been found in many animals ranging from various insects to zebrafish or mice (Stavenga 1992; Briscoe et al. 2003; White et al. 2003; Yoshimatsu et al. 2020; Szatko et al. 2020). Parts of the retina can also be specifically adapted for detecting mates, predators, water, food or host plants (Labhart and Nilsson 1995; van Hateren et al. 1989; Belušič et al. 2013; Perry and Desplan 2016; Heinloth et al. 2018; Meglič et al. 2019).

Diurnal butterflies are highly visual insects which interact with their colourful conspecifics, forage, oviposit, perch and patrol (Kinoshita and Stewart 2020). The visual sensory input is coming through their compound eyes, made of discrete optical units, the ommatidia. Each ommatidium contains nine photoreceptor cells that contribute their light-sensitive parts, the rhabdomeres, to a common light guide, the fused rhabdom (Arikawa 2017). The rhabdomeres are assemblages of tens of thousands microvillar membranes packed with rhodopsin molecules that absorb the photons guided through the rhabdom, triggering phototransduction (Hardie and Juusola 2015). The microvilli of single rhabdomeres are arranged with their long axes in a precise spatial pattern. Two cells (R1&2) have them aligned along the dorso-ventral axis and two cells (R3&4) along the horizontal axis. The nymphalid retina is not fully tiered (Nagloo et al. 2020), meaning that the microvilli of R1-8 are present at all depths, albeit in different proportions. The most pronounced tiering is found in R1&2, with rhabdomeres often present only in the distal retina. Cells R5-8 have the microvilli aligned along the diagonals, and the minute basal cell R9 is either aligned vertically, or has unaligned, curved microvilli (Arikawa 2003). The sensitivity of the photoreceptors to linearly polarized light is indicative of the angle of the microvilli: R1&2 are maximally sensitive to vertically, R3&4 to horizontally and R5-8 to diagonally polarized light (Arikawa and Uchiyama 1996). Thus, the angular maximum of the voltage response in intracellular recordings can be used to experimentally identify their cellular identity.

The photoreceptors in an ommatidium express different opsins with different absorbance spectra, which is the basis for their ability to mediate colour vision. In most nymphalid butterflies, the long wavelength-sensitive cells R3-9 express a single L opsin, maximally absorbing in the green or yellow part of the spectrum (Briscoe et al. 2003; Briscoe and Bernard 2005). These cells project short axons into the first optical neuropil, the lamina. These short visual fibres (SVFs) act as the main input into the achromatic pathway. Cells R1&2 project axons into the second neuropil, the medulla, and are termed as the long visual fibres (LVFs). Importantly, the SVFs project direct inhibitory synapses onto the axons of LVFs. The SVFs and LFVs together form colour-opponent photoreceptor pairs, providing the input for the neural circuitry mediating colour vision (Matsushita et al. 2022).

In the simple version of the retina (found e.g. in *Vanessa cardui* (Briscoe et al. 2003), *V. atalanta* (Zaccardi et al. 2006; Pirih et al. 2020), *Apatura ilia* (Pirih et al. 2022), butterflies from Nymphalini tribe (Briscoe and Bernard 2005)), cells R1&2, expressing either a U or B opsin (maximally absorbing in the ultraviolet or blue wavelength range, respectively), are inhibited by L receptors, and their sensitivity profiles can be described as U + G– or B + G– (Pirih et al. 2022). Opsin expression in these cells is stochastic, yielding three ommatidial types, named on the basis of the three R1&2 combinations (U/U, U/B and B/B) (Briscoe et al. 2003; Perry et al. 2016). In many nymphalid species, cells R1&2 can express an L opsin, which yields three additional ommatidial types (L/U, L/B and L/L) that add to the expanded retinal mosaic (McCulloch et al. 2022; Pirih et al. 2022). A study combining pupillary action spectra and electropyhsiologically determined spectral sensitivity of photoreceptors of a nymphalid *Charaxes jasius* has shown that the ommatidia with L-expressing R1&2 contain a red screening pigment that filters the light being propagated to the proximal part of the rhabdom (Pirih et al. 2022). Consequently, the basal R9 cells in these ommatidia are sensitive to red light; the R9 cells provide inhibitory input to the cells R1&2, which are thus best described as G + R– cells (Belušič et al. 2021).

A part of incident light that is not absorbed by the pigments is reflected back from the tracheoles at the base of the retina. Observing this light with an ophthalmoscope apparatus reveals the eye shine image (Stavenga 2002), the spectral composition of which depends on the tapetum reflectance and the absorbance of all pigments in the photoreceptor cells. The ommatidial types L/L, U/L, B/L that contain G + R– cells appear as dark-red due to the red screening pigment, while those containing U + G– and B + G– cells have no screening pigment and can appear whitish, green, yellow or orange, the exact colour depending on the ommatidial length (turning from green-yellow to red with increasing length) and the tuning of the tapetal multilayer (Pirih et al. 2022).

*Heliconius* butterflies have a complex retinal mosaic with red-shining ommatidia (McCulloch et al. 2016), which have the cellular toolkit for colour vision in the red part of the spectrum (Zaccardi et al. 2006). The complexity of the mosaic is in some species further increased by the duplication of U opsins (Briscoe et al. 2010) that enhances colour discrimination in the UV part of the spectrum for species and sexes that express the duplicate opsin, enabling a better detection of the wing patterns (Finkbeiner and Briscoe 2021; Chakraborty et al. 2023). The *Heliconius melpomene* genome contains four visual opsins, UV1, UV2, B and L, with UV1 and UV2 on different chromosomes (Dasmahapatra et al. 2012; Chakraborty et al. 2023). However, UV2 is not detectable in the *H. melpomene* retina at the protein level using anti-UV2 opsin antibodies (McCulloch et al. 2017). Furthermore, *H. melpomene* red-inhibited (Bg + R–, BG + R–, G + R–) cells co-express L and B opsins, but the role of the co-expression has not been studied yet (McCulloch et al. 2022). Here, we examine opsin co-expression using opsin labelling with antibodies, single cell recordings, and eye shine imaging. We show that B opsin expression is increasing from dorsal to ventral, gradually broadening the spectral sensitivity profile of L-expressing R1&2 in the short wavelength part. We discuss the implications of the modulated sensitivity for colour discrimination across the light field.

## Materials and methods

### Electrophysiology

Single cell recordings were performed in 20 specimens of *H. melpomene* rosina (10 males, 10 females), reared in a colony at UC Irvine, or purchased from an online reseller (Costa Rican Entomological Supply, Costa Rica). The animals were cold-anaesthetized on ice, immobilized and mounted on the side (right side to access the ventral retina of the left eye, left side for the dorsal retina of the right eye) in 1 ml plastic pipette tips with bees wax and mounted into a custom mini goniometric stage, precisely aligning the dorso-ventral axis with the horizontal plane for the recording. A 50 µm Ag/AgCl wire was inserted into the antenna as a reference electrode. The recording electrode was pulled with a P-2000 laser puller (Sutter, USA) from 1/0.5 mm outer/inner diameter borosilicate glass (Sutter, USA), loaded with 3 M KCl to yield 100–150 MΩ resistance and inserted into the eye with a micromanipulator (Sensapex, Finland) through a small hole in the cornea on the ventral or dorsal side of the eye, which was sealed with silicone vacuum grease. The signals were amplified with a SEC 10LX amplifier (NPI, Germany) and a CyberAmp 320 conditioner (Molecular probes, USA), digitized at 2 kHz sampling rate and stored in a PC with a Micro CED 1401 MK II (Cambridge Electronic Design, UK). Stimulation was provided with two light sources operating in iso-quantal mode, a multispectral LED array (Belušič et al. 2016) and an XBO arc lamp (Cairn, UK), filtered with a monochromator (B&M Optik, Germany) and a continuously graded neutral density filter (Thorlabs, Germany), both motorized and controlled with Due microcontrollers (Arduino, Italy). The two beams were combined with a 50% polka-dot beamsplitter (Thorlabs, Germany) and projected co-axially through a focusable objective with the aperture and field diaphragms in the optical pathway to shape the beam. The recorded ommatidium was aligned with the beam and photoreceptor cell impalement was recognized by the sudden voltage drop to the resting membrane potential and depolarizing responses to light flashes. Every cell was quickly (2 s) scanned for spectral sensitivity with the LED array and analysed further if necessary. Spectral sensitivity was scanned in detail with the monochromator from 300 to 700 nm and back in 5 nm steps. Polarization sensitivity was measured with a motorized rotating, UV-capable polarizer (Bolder Optik, USA) inserted into the beam. Spectral and polarization sensitivity were calculated via reverse Hill transformation (see e.g. Belušič et al. 2016), with Hill function parameters derived from the intensity run, recorded at peak sensitivity wavelength between 4 and 0 OD (optical density units) in 0.2 OD increments (Pirih et al. 2022). For selective adaptation experiments using blue (~ 450 nm) or green light (~ 550 nm), spectral sensitivity was recorded either by scanning with the LED array and constantly adapting with light from the monochromator, or vice versa—in the latter case, the LED array allowed us to use double selective adaptation, as shown in Figure two.

### Immunohistochemistry

Methods were adapted from previous studies (Hsiao et al. 2012; Perry et al. 2016; McCulloch et al. 2017; Chakraborty et al. 2023). Butterflies were purchased from online seller (Costa Rica Entomological Supply, Costa Rica) as pupae and housed in a greenhouse after eclosion. The specimen was collected from the greenhouse and euthanized via rapid thorax crushing and decapitation. In cold 1 × PBS and under a dissecting scope, the head was then bisected and excess tissue excised. The individual eyes were quickly fixed in 4% paraformaldehyde for 30 min with rotation in a cold room (~ 15 °C) and then underwent step-wise sucrose baths (10, 20, and 30% sucrose in 1 × PBS) for two hours with rotation in a cold room (~ 15 °C). The corneal lens was carefully removed, and the eyes were embedded in gelatin-albumin. The gelatin-albumin blocks then underwent 15 h of post-fixation in 4% formalin at 4 °C. Afterwards, they were sliced using a Compresstome V4-310-0Z (Precisionary, USA) to 80 µm and stained. The slices were blocked with 5% (v/v) normal donkey serum in 0.3% PBST (Triton-X in 1X PBS) for one hour at room temperature. Then slices were incubated overnight at 4 °C with primary antibodies: 1:50 anti-UV1, 1:200 anti-blue, and 1:150 anti-LW. Afterwards, slices were washed five times with 0.3% PBST for 15 min each wash. Slices were then incubated overnight at 4 °C with secondary antibodies: 1:250 AlexaFluor 647 donkey anti-guinea pig, 1:250 AlexaFluor 488 donkey anti-rat, and 1:250 Cy3 donkey anti-rabbit. Slices were again washed five times with 0.3% PBST for 15 min per wash and then mounted in 70% glycerol (in 1 × PBS). Images were taken using a LSM 980 Airyscan 2 (Zeiss, Germany) confocal microscope and exported using ZenBlue 3.5 (Zeiss, Germany). Images were then analysed using LabPlot2 and RStudio.

### Eyeshine

Eyeshine was recorded using a custom epi-illumination tele-microscopic setup equipped with two XYZ-stages of micrometre resolution and a motorized goniometric stage that allowed for precise positioning of the specimen within the beam path and the focus of the objective (Pirih et al. 2020). A custom-made LED array light source was used for the recordings (Belušič et al. 2016). The experiments were done in fresh, living, immobilized butterflies (7 males, 5 females), positioned with the eye in the centre of rotation of the goniometric stage, which facilitated the mapping of the eyeshine along arbitrary elevation and azimuth. A (0°, 0°) indicates a frontal position, i.e. head of the animal oriented such that it faced directly into the objective, which was ensured by aligning the back of the eyes parallel to the objective lens. Negative and positive elevations indicate ventral and dorsal directions, respectively. Generally, each specimen was moved in elevation steps of 30°. The size of the step was determined experimentally and chosen because it offered the best trade-off between imaging new parts of the eye and retaining sufficient ommatidial overlap between images, i.e. it allowed for stitching the ommatidial lattice across the entire eye.

When viewed by the human observer, the ommatidia appeared as orange-red or dark red. To enhance the contrast between the two classes, hyperspectral images of the eyeshine were mapped into RGB space as follows. A series of grayscale images of the dark-adapted eyeshine and light-adapted background evoked with monochromatic light from the LED array (675, 653, 635, 622, 610, 594, and 575 nm), was obtained with a monochrome camera (Blackfly, Teledyne, Canada) at each elevation angle. The images were processed using Fiji 1.54p (Schindelin et al. 2012). The background images were first filtered using a Gaussian filter (sigma = 1) and then subtracted from their respective dark-adapted counterparts. This removed the imaging artefacts such as the internal lens reflections. The cleaned monochromatic images were grouped according to the observed eyeshine pattern, averaged by taking their median pixel intensity, and then mapped into the RGB colour space as shown in Fig. 6A. Specifically, the images obtained at 675 and 653 nm were mapped to the R channel, while those obtained with 635, 622, 610, and 594 nm were mapped to the G channel and B channel was left empty (in Fig. 6A) or to all channels (thus appearing white, Fig. 6B).

### Analysis of the eyeshine maps

The number of red and non-red ommatidia were counted at each elevation angle. First, a segmentation mask was created using a custom-made python script based on the ommatidial detecting algorithm (ODA; Currea et al. 2023). This detected all ommatidia within image, without differentiating between the different types. The subset of segmentation masks and the RBG images (15 out of 170) was used to train an object classification model in Ilastik (1.4.0, Berg et al. 2019) that was afterwards applied to the entire dataset. The model was trained to differentiate between red, non-red, and dark ommatidia. The latter lacked any intensity information and never appeared bright in any of the eyeshine images. The results were exported as labelled masks, each label corresponding to a particular ommatidial type. The different objects (ommatidia) in such masks were counted, divided by the total number of ommatidia per image, and averaged per elevation and sex to compare their abundance at different locations along the dorso-ventral axis (Fig. 6C).

## Results

**Fig. 1:**
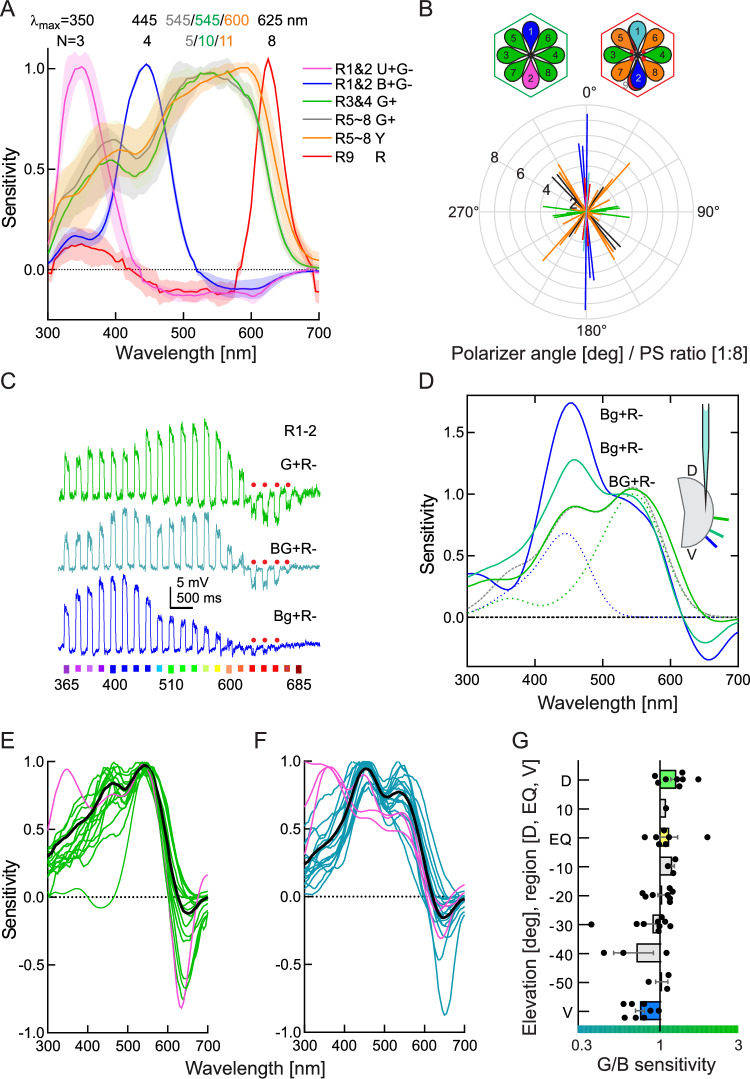
Electrophysiological analysis of *H. melpomene* retina. **A** Spectral sensitivity (mean ± SEM) of all photoreceptors (except G + R– R1&2, which show a dorsal–ventral gradient of blue-green sensitivity). **B** Polarization sensitivities of the photoreceptors in (**A**); maximal sensitivity angles are approximately parallel to microvillar orientations schematized in top insets, depicting ommatidia according to their colour in the eyeshine (red and non-red); cells R1&2 express either U (pink) or B (dark blue) opsin (left, non-red type), or L opsin (right, red type); R5-9 in both types of ommatidia express L opsin, but filtering with the red perirhabdomal pigments shifts the sensitivity of R5-8 and R9 to orange and red, respectively. **C** Voltage signals and **D–F** spectral sensitivities of BG + R– and Bg + R– R1&2; **C**, **D**, cells recorded along a single dorso-ventral microelectrode excursion in an eye, positions indicated in inset in (**D**). Dotted and dashed curves in (**D**) are absorbance templates of opsins, peaking at 445 nm (dotted blue), 545 nm (dotted green) and the sum of 445 nm + 545 nm templates, fitted to the G + R– cell (dashed grey). **E** Green-sensitive R1&2 dorsally, **F** ventrally. Pink curves show spectral sensitivity of UV-peaking cells with red opponency; black curves, average sensitivity. **G** Green vs. blue sensitivity ratio of cells in (**E**, **F**), recorded at different positions along the dorso-ventral axis, indicated with degrees or D dorsal, EQ equatorial, V ventral. Cells peaking in the UV are not included in the analysis and in the average curves in (**E**, **F**)

Individual photoreceptors in *Heliconius* compound eyes were analysed with microelectrode recordings and classified based on their spectral sensitivity peaks (λ_max_) and angular maxima (*φ*_max_) of sensitivity to polarized light (Fig. 1A, B). Cells, maximally sensitive to vertically polarized light (i.e. to photons with e-vector parallel to the dorso-ventral eye axis) were classified as the long visual fibre photoreceptors (LVFs) R1&2. Two classes of R1&2, maximally sensitive to ultraviolet (UV) or blue (B) light and inhibited by long-wavelength photoreceptors (G–), were labelled as U + G– and B + G–, respectively. As previously demonstrated in *Charaxes jasius* (Pirih et al. 2022), these cells (in combinations U + G−&U + G−, U + G−&B + G−, B + G−&B + G−) presumably reside in the non-red ommatidia without the red screening pigment, together with cells, maximally sensitive to green (G) light, polarized horizontally (R3&4) or diagonally (R5-8). In red ommatidia, at least one R1&2 expresses L opsin, R5-8 have a red-shifted spectrum and peak in the yellow (Y), while the basal cell R9 is the red receptor (R) (Belušič et al. 2021), which expresses the same L opsin as R3-8 (Briscoe 2008). Its sensitivity could be recorded indirectly, with selective chromatic adaptation of red-inhibited R1&2 with green light. Incomplete selective adaptation resulted in apparent green inhibition of R9 (Fig. 1A, red curve). We found no differences between the sexes, therefore the results from males and females are pooled together.

The retinal architecture of *Heliconius* is similar to the other nymphalid butterflies with the complex, expanded retinal mosaic—in addition to the non-red ommatidia with U + G– and B + G– LVFs, the retina also contains red ommatidia with green-sensitive R1-2, inhibited by the red receptors (G + R–). The spectral sensitivity profile of these cells gradually changed along the dorso-ventral axis (Fig. 1C–G). Ventrally (from the ventral eye edge to ~ −30° latitude), their peak was in the blue (Bg + R–; lowercase g indicates low sensitivity in the green vs. high in the blue); the blue peak decreased towards the eye equator (−20° to + 10° latitude), where both peaks were about equal (BG + R–). In the dorsal retina (above 10°), the blue peak gradually diminished (G + R–; uppercase G and absence of B indicate a larger sensitivity peak in the green). Spectral sensitivity of red-inhibited cells could be fitted in the 400–600 nm range with a linear sum of two rhodopsin absorbance templates (Stavenga et al. 1993), with fixed absorbance peaks at 445 and 545 nm and relative amplitudes A_445_ and A_545_ as free parameters (Fig. 1D). The cells were then categorized according to the ratio A_445_/A_545_: Bg + R– if A_445_/A_545_ > 1.1, BG + R– if 0.9 < A_445_/A_545_ < 1.1, G + R– if A_445_/A_545_ < 0.9. Additionally, we encountered red-inhibited cells with another sensitivity peak in the UV (Fig. 1E, F).

**Fig. 2:**
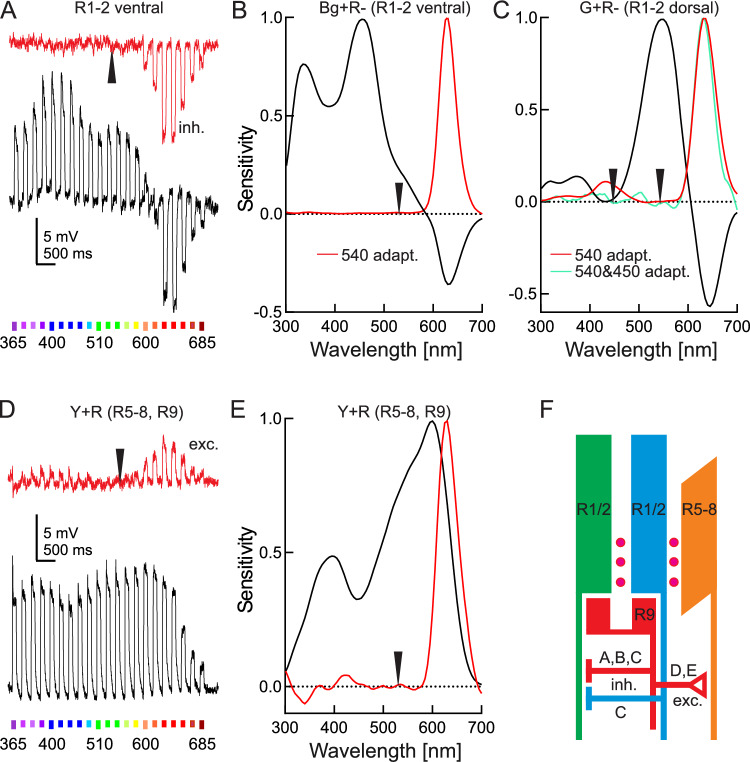
Variations of spectral sensitivities due to electrical interactions. **A** Voltage responses to spectral flashes and **B** spectral sensitivity of a Bg + R– R1&2 cell in the ventral retina, dark-adapted (black trace) and green-adapted (red trace). **C** Spectral sensitivity of dark-adapted (black curve), green-adapted (red curve), green-and-blue-adapted (turquoise curve) G + R– R1&2 cell in the dorsal retina. **D** Voltage responses to spectral flashes and **E** spectral sensitivity of an R5-8 cell, dark-adapted (black trace) and green-adapted (red trace). **F** R9 inhibits BG + R– R1&2 and excites R5-8. Dorsal G + R– R1&2 is inhibited both by R9 and B-expressing B + G- R1&2. Magenta dots indicate the red screening pigment. Arrows in (**A–E**) indicate the wavelength of adapting light

As in previous studies (Belušič et al. 2021; Pirih et al. 2022; Ilić et al. 2022), the spectral response of R9 cells in red ommatidia could only be inferred through recordings from G + R– cells using green adapting light that isolated the inhibitory responses of the colour-opponent red unit (Fig. 2A), yielding an estimate for the spectral sensitivity of R9 (Fig. 2B). Interestingly, the same selective adaptation protocol, applied in the dorsal G + R–, revealed that the isolated response of the opponent units contained an additional sensitivity peak in the blue, presumably arriving via an inhibitory synapse from the blue receptors. The blue signal could be finally suppressed with double band, green + blue selective adaptation (Fig. 2C). Lastly, selective adaptation of Y receptors (red-shifted R5-8 cells) with green light isolated the response of the red receptors, but with positive polarity (Fig. 2D, E). This indicates that the basal R9 in red ommatidia forms an elaborate network which includes both inhibitory synapses to G + R–, BG + R– and Bg + R– cells as well as excitatory synapses to Y cells. In the red ommatidia in the dorsal part of the eye, the G + R– cells are additionally inhibited by blue-sensitive cells, probably R1&2 (Fig. 2F).

We currently do not have the anatomical data to state whether R9 in *H. melpomene* is a SVF or LVF. However, serial EM sections of the lamina of *A. paphia* (belonging to the same family, Nymphalidae: Heliconiinae) show that R9 (inhibition visible in Fig. 7B) in all segmented ommatidia is a short visual fibre (manuscript in preparation).

**Fig. 3:**
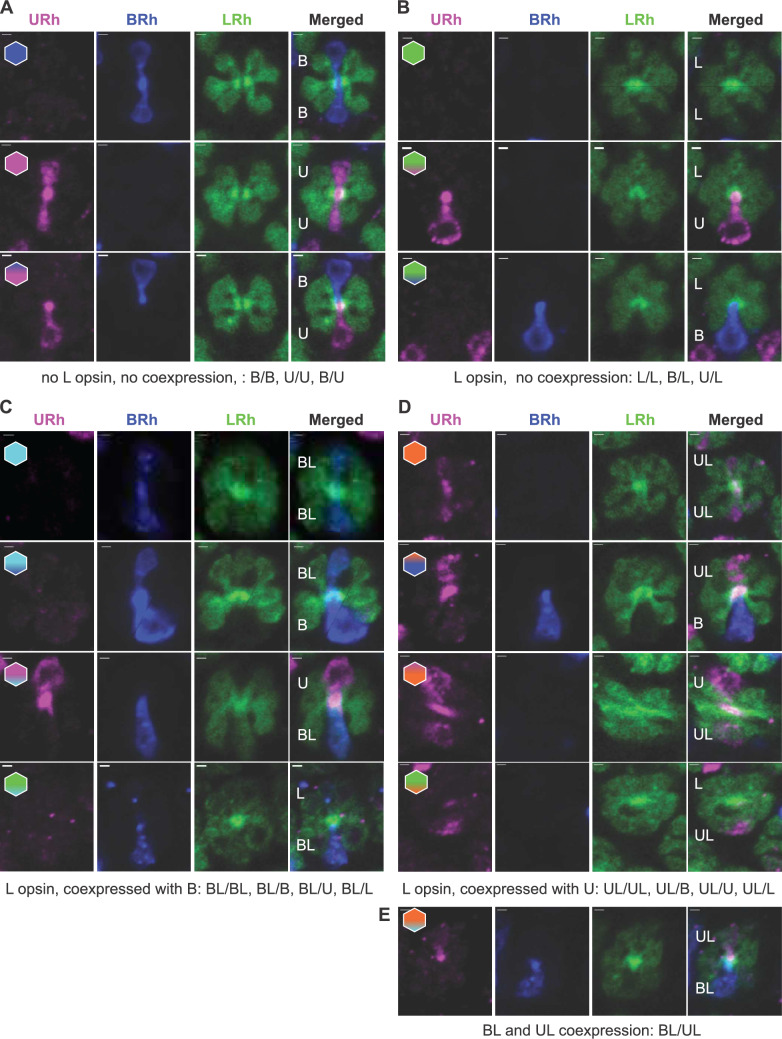
Immunohistochemical classification of ommatidial types. Ommatidia sorted according to opsin expression in R1&2 (vertically oriented cells); section labelled with anti-rhodopsin (Rh) antibodies against UV (magenta channel; U; URh), blue (blue channel; B; BRh) and green-absorbing (green channel; L; long wavelength, LRh) opsins. **A** R1&2 expressing either U or B opsin **B–E** R1&2 expressing L opsin alone (**B**) or together with B (**C**) or U (**D**) opsin. Hexagonal schemes indicate ommatidial type. Scale bars, 2 µm

The electrophysiological results led us to hypothesize that the BG + R– and Bg + R– cells in red ommatidia co-express the B and long-wavelength (L) opsins, with the highest degree of B opsin expression ventrally, decreasing towards zero dorsally; occasionally, these cells peaked in the UV, indicating that they might coexpress U and L opsins. We analysed opsin expression pattern in the retinal cross sections that were labelled with antibodies against U, B and L opsins (Fig. 3). Cells R3-8 were stained exclusively with anti-L antibodies, while cells R1&2 were stained either exclusively with anti-U, anti-B, anti-L, or co-stained with anti-U and anti-L, or anti-B and anti-L antibodies. In individual ommatidia, cells R1&2 occurred in all combinations typical for the basic nymphalid retina (U/U, U/B, B/B; Fig. 3A), or in combinations with L-opsin expressing R1&2 (Fig. 3B–D), typical for the complex nymphalid retina (U/L, B/L, L/L; Fig. 3B). L and B opsin co-expression in R1&2 yielded four more combinations (BL/BL, BL/B, BL/U, BL/L; Fig. 3C), similarly also L and U co-expression (UL/UL, UL/B, UL/U, UL/L; Fig. 3D). Lastly, the fifteenth ommatidial type was found, where one R1&2 cell co-expressed U and L, and another co-expressed B and L opsins (Fig. 3E; UL/BL).

**Fig. 4:**
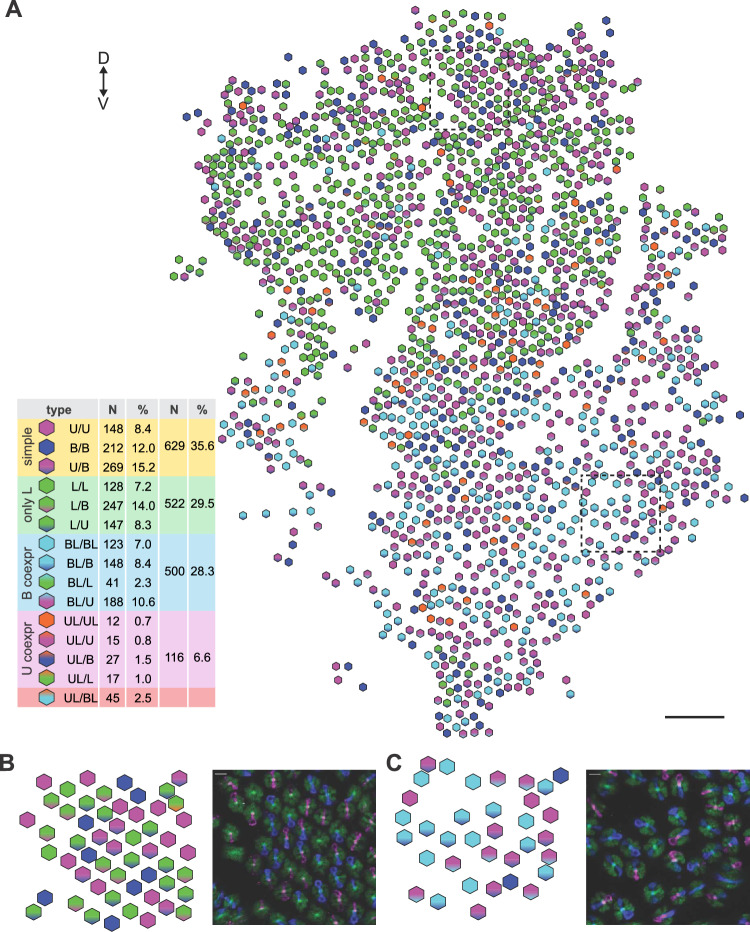
Rhodopsin expression pattern across the retina. **A** Entire section with identified ommatidial types; dashed squares indicate parts, analysed in (**B**, **C**); dorsal and ventral directions indicated at the top left. Total number of ommatidia = 1767. Table in A shows numbers and percents of individual ommatidial types and types, grouped according to L expression and coexpression. U = magenta; B = blue; L = green channel. Scale bars: A, 200 µm, B and C, 20 µm

The different ommatidial types were localized in a large cross-section of the retina, which contained 1767 ommatidia (Fig. 4A). The ommatidia, constituting the basic retinal mosaic (U/U, U/B, B/B), represented about one third of all ommatidia, while those containing L-expressing R1&2 and constituting the expanded retina, represented about two thirds of all ommatidia (Table in Fig. 4A). The ommatidia were distributed semi-randomly, with the dorsal side enriched with cells expressing only L opsin (labelled green; Fig. 4B) in R1&2, and the ventral side enriched with cells co-expressing L and B opsin (labelled turquoise; Fig. 4C) in R1&2. In contrast to the continuously graded sensitivity to blue or green light in L-expressing R1&2 observed in electrophysiological recordings, here the cells appeared as either positively or negatively reacting to the antibodies, because the immunohistochemical labelling and sample imaging are too coarse to reveal the fine gradation.

**Fig. 5:**
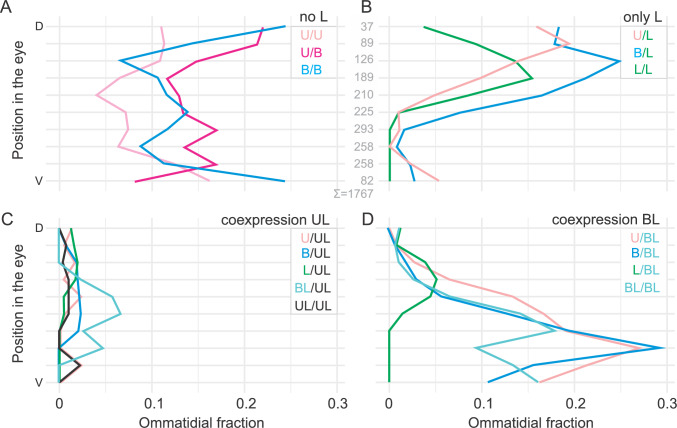
Ommatidial type densities along the dorso-ventral axis; ommatidia are labelled according to opsin expression (U, B, L) and co-expression (UL, BL) in R1&2. The section shown in Fig. 3 was divided into 10 horizontal strips of equal width. **A** Ommatidia not expressing L opsin in cells R1&2. **B–D** Ommatidia expressing L opsin in cells R1&2. Ommatidia expressing only L opsin (**B**), co-expressing L & U opsins (**C**) and co-expressing L and B opsins (**D**). D, dorsal, V, ventral. Numbers between A and B indicate total ommatidial counts (N) per strip (N = A + B + C + D)

The slice shown in Fig. 4 was then divided into ten horizontal strips of equal width. In each strip, the different ommatidial types were counted and their number was normalized to the total count in each strip (counts shown in Fig. 5B), yielding the spatial distribution of ommatidial type densities across the dorso-ventral axis (Fig. 5). The ommatidia without L opsin-expressing R1&2 (U/U, U/B, B/B; non-red ommatidia; Fig. 5A) were more frequent in the dorsal strips. The ommatidia with R1&2 expressing exclusively L opsin were located mostly in the dorsal half (Fig. 5B), while the LB-co-expressing R1&2 were located mostly in the ventral half of the slice (Fig. 5D). We note that the reliability of ommatidial fraction estimates decreased at the ventral and dorsal edges due to lower numbers of ommatidia. The UL-co-expressing R1&2 were rare, and a spatial gradient could not be determined (Fig. 5C).

**Fig. 6:**
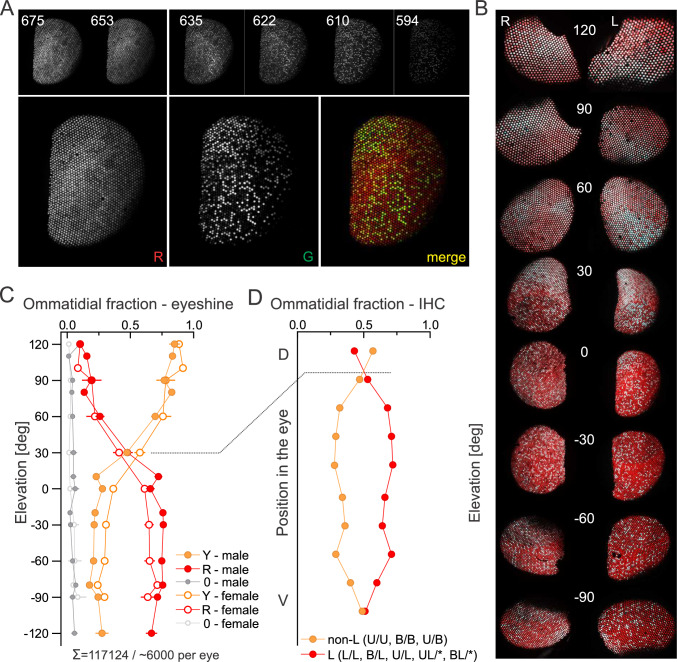
Distribution of ommatidial types across the eye. **A** Eyeshine evoked with monochromatic light (wavelengths in nm indicated in images); 2 far-red and 5 yellow–red images were overlaid, contrast-adjusted and merged into a false-coloured image showing red and non-red ommatidia. **B** Eyeshine, showing red (red) and non-red (white) ommatidia, mapped along the dorso-ventral axis. **C** Fractions of red (R), non-red (Y) and dark (0) ommatidia along the dorso-ventral axis, imaged in 19 eyes of 12 butterflies (7 males, 5 females; mean ± S.D.). **D** Fractions of ommatidia with R1&2 expressing either only U or B (non-L) or L alone or with U and B (L, BL, UL) opsins, data from Fig. 3. Dashed line connects the locations in the eyeshine and in the labelled section, where the ratio R:nonR is equal

To localize the analysed fragment of the retina within the eye, we imaged the eyeshine and classified the ommatidia as red and non-red (Fig. 6A). We assumed that the non-red ommatidia were also 'non-L', i.e. those that do not contain an L opsin-expressing R1&2 (U/U, U/B, B/B), while the red ommatidia belong to the remaining 12 ommatidial types that contain at least one L opsin-expressing R1&2 and could thus be labelled as 'L'. The eyeshine was mapped across the dorso-ventral axis and the ommatidial fraction was determined for each image stack (Fig. 6B, C). At the dorsal and ventral extremes, the fraction of red ommatidia was ~ 0.1 and ~ 0.6, respectively. The two fractions were about equal at 30° above the eye equator. In the immunohistochemically labelled retina, the two fractions were about equal in the second strip from the dorsal side (Fig. 6D), which allowed us to locate the labelled slice into the eye centre. Equal fractions were also found in the ventral most strip, but we assume that this was an artefact due to the low count.

Taken together, the optically, immunohistochemically and electrophysiologically determined regionalization of the *Heliconius* compound eye reveals the intricate, multi-levelled fine tuning of the retinal mosaic.

## Discussion

We have revealed that in *Heliconius melpomene*, the long visual fibre photoreceptors R1&2, likely residing in the ommatidia containing the red screening pigment, co-express the L opsin together with the B opsin, and, in a small fraction, with the U opsin. The cells co-expressing L and B opsins receive inhibitory inputs from the basal R photoreceptors and seem to be particularly suitable for encoding blue-green vs. red colour contrasts. Opsin co-expression has been previously found in various insects (Hu et al. 2014; Schmeling et al. 2014) including butterflies from different families and has been assumed to be a mechanism for creating photoreceptors with broadened sensitivity (Arikawa et al. 2003; Sison-Mangus et al. 2006; Ogawa et al. 2012) and a developmental phenomenon accompanying photoreceptor differentiation (Arikawa et al. 2017). In cichlid fish, graded opsin co-expression fine-tunes the spectral sensitivity by maximizing the background photon catch and improves colour discrimination across the visual field (Dalton et al. 2014, 2015, 2017). Similarly, the co-expression of B and L opsin in the photoreceptors of *Heliconius* is likely graded across the visual field and results in a fine tuning of the spectral sensitivity. The spectral sensitivity of the cells is predominantly narrow-band green in the part of the retina which views the directions above and around the horizon (G + R–), broad-band blue-green just below the horizon (BG + R–), and blue-peaking in the downward-looking ventral part of the retina (Bg + R–). Owing to opsin co-expression, the BG + R− and G + R− receptors might get roughly equally depolarized when viewing the green and blue-rich ventral hemisphere and skyline, respectively (Fig. 8). However, this assumption should be supported by a meticulous analysis of spatio-spectral structure of butterfly habitats and imaging of head and eye position across different locomotory states (Bergman et al. 2021). Furthermore, the existing environmental imaging data do not show a continuous spectral or intensity gradient in the ventral hemisphere, which could be matched by the graded opsin co-expression (Nilsson and Smolka 2021). Additional factors which could favour the evolution of graded opsin co-expression include (but are not limited to) the variable thickness of the retina and the associated R1&2 rhabdomere length, which determine the photon catch and complex effects of self-screening in the rhabdoms (Laughlin et al. 1975). Lastly, the blue opsin, present in the distal rhabdom, could augment the filtering effects of the red screening pigment, thereby rendering the basal red receptors less sensitive to short-wavelength light and thus improving colour discrimination.

**Fig. 7:**
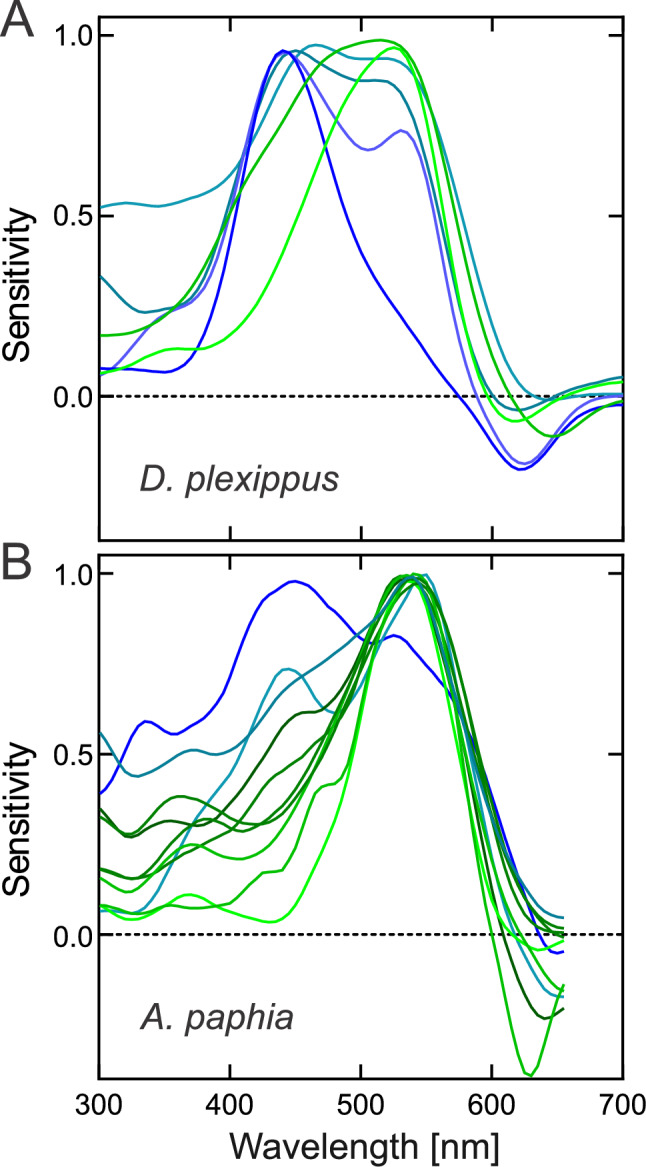
Spectral sensitivities of BG + R– R1&2 photoreceptors along the dorso-ventral axis in **A** *Danaus plexippus* and **B** male *Argynnis paphia*

**Fig. 8:**
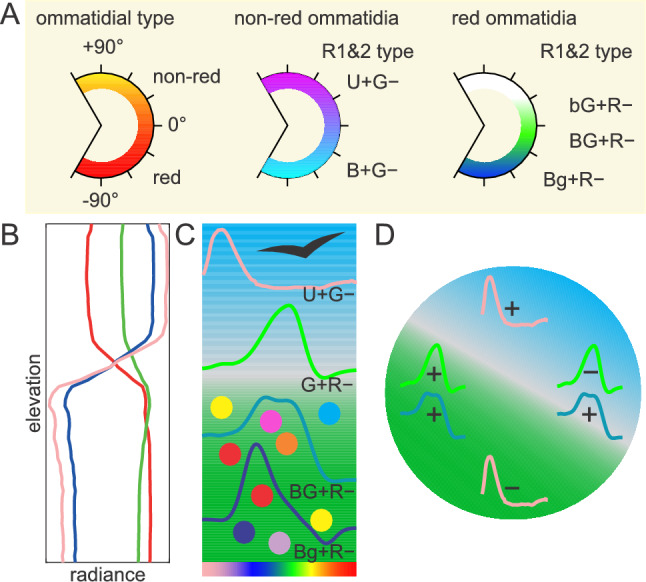
Spectral tuning of the retina to the light field. **A** Multi-layered gradation of ommatidial and photoreceptor types along the dorso-ventral axis. **B** Intensity gradient in the UV, blue, green and red spectral bands for open terrain (adapted from Nilsson and Smolka 2021; x-axis logarithmic). **C** Long visual fibre photoreceptors are matched to the spectral contrasts in the light field; dark objects are viewed against the UV-rich, decluttered sky (shown in blue); colour-rich targets inhabit the ventral hemisphere (shown in green); progressive decrease of blue light is compensated by increasing B co-expression with L opsin. **D** Possible role of the narrow-band G + R− and broadband BG + R– receptors in horizon segmentation (of sky represented by blue and ground indicated by green); + and – indicate photoreceptor excitation and inhibition, respectively

The finding is not limited to *H. melpomene*; we observed a similar dorso-ventral tuning in *Danaus plexippus* (Nymphalidae: Danaini) and in the males of *Argynnis paphia* (Nymphalidae: Heliconiini) (Fig. 7), but the precise spatial distribution of opsin proteins in those butterflies has yet to be determined. The three species live in very different visual environments. *H. melpomene* inhabits the tropical forests of Central and South America, and is mostly found in sunny, open patches (personal observation from Fr. Guyana); *D. plexippus* can be found in the open fields in the United States and overwinters in the forests of Central Mexico; *A. paphia* patrols along forest edges across Eurasia (Fig. 8).

The retina in all three species appears to be a matched filter, optimized to the gradients of the light field in their visual habitats. However, the spectral and intensity gradients which could be common to the terrestrial habitats in which these species live, are not so obvious. The light fields are enriched with UV and blue light above the horizon, and with green light in the lower hemisphere, while the intensity is varied by about 1 log unit, with the maximum (excluding direct sun) often at the horizon due to light scattering in the haze. Objects, superimposed on the bright sky background, e.g. predatory birds are best detected with U + G– photoreceptors that detect dark spots on a background, which is uncluttered in the UV (Belušič et al. 2013; Franke et al. 2024). The colourful objects in the terrestrial hemisphere are superimposed on the more cluttered background composed of green vegetation, soils, rock and sand that mostly reflect well in the far-red spectral range, and water bodies with variable spectral composition. In front of a green background, the colour contrast is the largest when the objects are non-green, i.e. UV, blue, red or purple (blue + red, UV + red). These are often the colours of flowers, fruits and butterfly wing patterns. Photoreceptors encoding non-red (broad blue-green) vs. red colour contrast, such as Bg + R– and BG + R–, are certainly valuable in all these scenarios.

Interestingly, the G + R– receptors around and above the eye equator are blind to blue due to the absence of B co-expression and inhibition by blue cells. This might be specific to blue receptor-containing B/L ommatidia, which are the most frequent ommatidial class at the eye equator. In combination with other LVFs (B + G–, U + G–), they can be used to view and segment the horizon during flight. This task is presumably performed by insect ocelli (Stange et al. 2002), which are greatly reduced in diurnal butterflies, so that retinal specializations for flight stabilization should not be too surprising.

The recordings revealed an intricate synaptic network, which also includes the R9 excitatory coupling to R5-8. This mechanism, probably together with the rhabdomal filtering with red pigments, slightly red-shifts the sensitivity of Y cells, and improves the signal-to-noise ratio by a very small fraction. The subtle evolutionary advantages due to synaptic tuning and opsin co-expression are difficult to appreciate without experimental manipulation in behaving butterflies and comprehensive computational modelling of retinal mosaic connectivity and performance in various scenarios.

## Data Availability

No datasets were generated or analysed during the current study.
